# Histiocytic Sarcoma in a Kidney Transplant Patient: A Case Report and Review of the Literature

**DOI:** 10.1155/2016/3591050

**Published:** 2016-10-03

**Authors:** Maressa Pollen, Siraj El Jamal, Jack Lewin, Varsha Manucha

**Affiliations:** Department of Pathology, University of Mississippi Medical Center, Jackson, MS, USA

## Abstract

*Objective*. Histiocytic sarcoma (HS) is an aggressive neoplasm with only limited number of reported series of cases and rare case reports of occurrence as a posttransplant neoplastic disorder. The etiology and pathogenesis of the disease is unknown and the optimal treatment is still under investigation. We describe an unusual case of HS in a patient with a remote history of kidney transplant.* Method and Results*. A 54-year-old male with a remote history of renal transplantation under maintenance immunosuppression presented with features of sepsis. CT abdomen revealed multiple heterogeneous masses in bilateral native kidneys and liver and enlarged abdominal and retroperitoneal lymph nodes. Viral serology work-up was negative. Needle core biopsy revealed a highly undifferentiated neoplasm comprised of highly atypical large cells with eosinophilic to vacuolated cytoplasm and hemophagocytosis. Extended panel of immunohistochemistry proved histiocytic lineage for the tumor cells. The patient expired 2 weeks following the diagnosis.* Conclusion*. Our case along with three previously published case reports raised the possibility of HS as a treatment-related neoplasm or a posttransplantation neoplastic disorder in solid organ transplant recipients.

## 1. Introduction

Posttransplant lymphoproliferative disorder (PTLD) is a recognized complication arising in allograft recipients treated with immunosuppressive drugs with a reported incidence of 1-2% in renal transplant patients [1]. PTLD may occur, even many years after transplantation, and is comprised of a histologic spectrum, ranging from hyperplastic-appearing lesions to frank non-Hodgkin's lymphoma or multiple myeloma histology and recently T-cell lymphomas. Herein we present a case of histiocytic sarcoma arising in the native kidney of a patient with a history of remote renal transplantation. Histiocytic sarcoma is a rare and often aggressive neoplasm with only a limited number of reported cases. To the best of our knowledge there are only 3 case reports on a kidney transplant recipient [2–4]. The current epidemiology estimates that less than one percent of tumors presenting in soft tissue or lymph nodes can be defined as histiocytic sarcoma (HS) [5]. The pathognomonic attributes of this tumor remain elusive even though several studies have been published in attempts to characterize reliable phenotypic and genotypic features including associations with germ cell tumors and with malignant lymphoma [5]. Our case along with the three other published case reports of HS in renal transplant recipients raised the possibility of HS being one manifestation of a late posttransplantation lymphoproliferative disorder (PTLD).

## 2. Case Report

**Figure 1:**
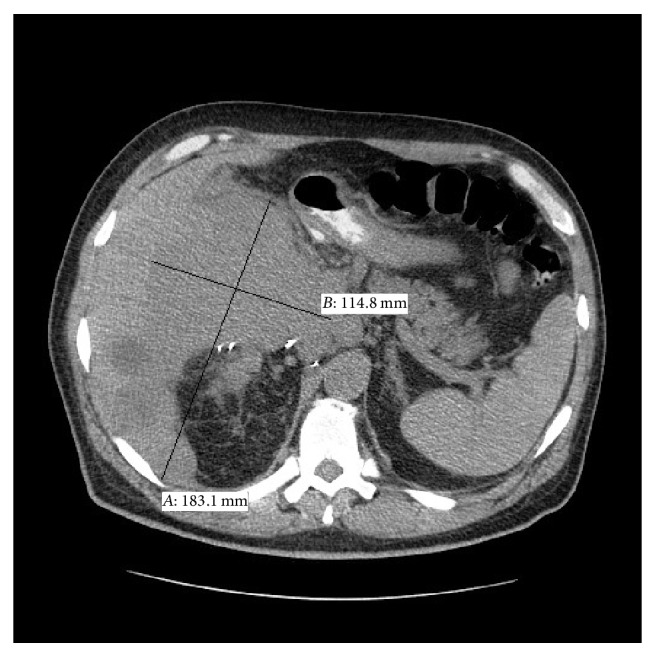
CT image showing involvement of kidney and liver by the tumor.

**Figure 2:**
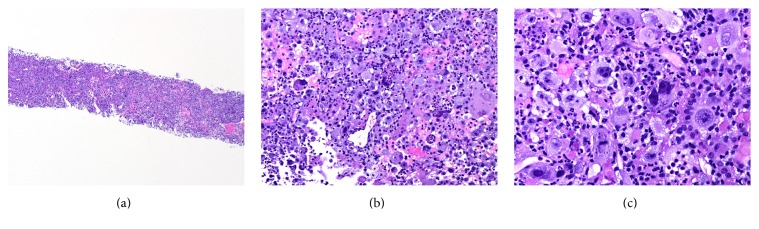
(a) Hypercellular cores comprised of sheets of noncohesive large tumor cells and neutrophils infiltrating renal tubules (H&E, 40x). (b) Tumor cells engorged with neutrophils and intimately juxtaposed with renal tubular epithelium (H&E, 400x). (c) Pleomorphic tumor cells with abundant eosinophilic to vacuolated cytoplasm, coarse nuclear chromatin, and multiple prominent eosinophilic nucleoli (H&E, 600x).

A 57-year-old man, with status postremote (18 years prior) renal transplant due to chronic kidney disease and hypertensive nephropathy, presented with complaints of fever, fatigue, and decreased appetite with concomitant 40-pound weight loss over the previous three months. At the time of admission the patient's concurrent medical problems included gout and hypertension. His medications list included antihypertensive agents, xanthine oxidase inhibitors, prednisone, cyclosporine, and mycophenolate. Laboratory investigations upon arrival revealed a neutrophilic predominant leukocytosis, 89% (ref. 44–65%) of total white blood cells with an accordingly low lymphocyte proportion, 3% (ref. 25–46%). Microbiology results were noncontributory and were reported negative for the following: EBV, BK virus, CMV, histoplasmosis, legionella, influenza A and influenza B, aspergillus, and MRSA. Blood and urine cultures were similarly negative. Imaging studies revealed an 18 cm heterogeneous mass involving the liver as well as multiple masses involving the native kidneys bilaterally (Figure 1). Of note a 6 cm mass with a calcific, thickened wall arose from the lower pole of the native right kidney and was suspected to be the primary diagnostic lesion. A diagnosis of septic shock and likely underlying posttransplant lymphoproliferative disease was rendered. A CT-guided needle core biopsy was performed. Touch imprints of the cores at the time of on-site evaluation revealed large atypical histiocytoid cells engorged with degenerating inflammatory cells. An infectious pathology could not be excluded. The needle core biopsy comprised of highly cellular cores almost entirely replaced by sheets of noncohesive large cells was intimately juxtaposed with renal tubular epithelium, partially destroying and replacing the tubules (Figure 2(a)). Tumor cells were pleomorphic, 3-4 times the size of tubular epithelial cells, with abundant eosinophilic to vacuolated cytoplasm, round nuclei with coarse chromatin, and multiple prominent eosinophilic 1-2 nucleoli (Figures 2(b) and 2(c)). Large multinucleated forms were also seen. Many of these large cells were engorged with nuclear debris and degenerating inflammatory cells. Increased number of neutrophils were seen intermixed with the tumor cells to the point of obscuration of tumor cells. Scattered atypical mitotic figures and areas of necrosis were identified.

**Figure 3:**
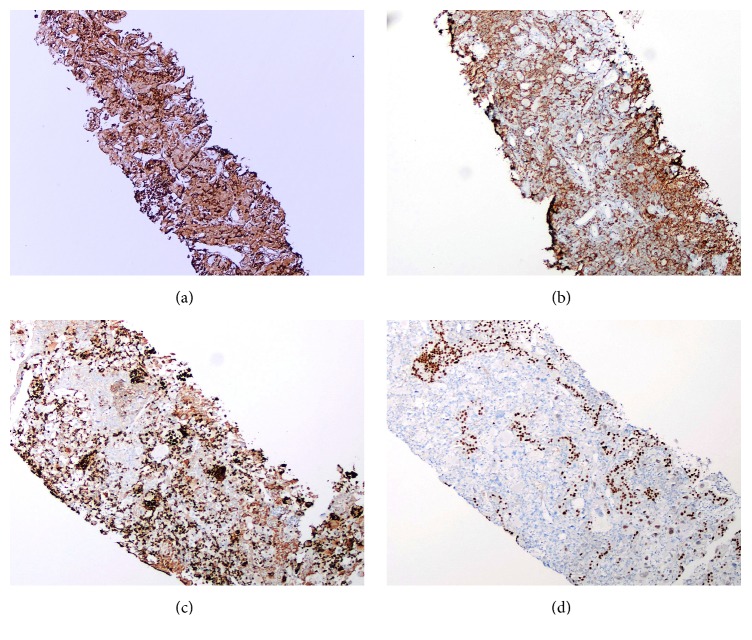
(a) Immunohistochemistry for CD68 highlights the tumor cells (100x). (b) Immunohistochemistry for lysozyme shows granular positivity in the cytoplasm of the tumor cells (100x). (c) Immunohistochemistry for HAM56 shows granular positivity in the cytoplasm of the tumor cells (100x). (d) Immunohistochemistry for PAX8 highlights the nuclei of renal tubular epithelial cells (100x).

A large panel of immunohistochemical stains were performed in order to rule out other large cell neoplasms such as large cell lymphoma, melanoma, and carcinoma. Tumor cells were positive for CD68 (Figure 3(a)), lysozyme (Figure 3(b)), HAM 56 (Figure 3(c)), and CD4. Tumor cells were negative for LCA (Cd45), myeloperoxidase, CD21, CD23, Cd1a, Cd3, CD20, CD56, CD99, broad spectrum keratins, EBV/LMP-1, and S-100. The renal tubular epithelial cells were highlighted by CK7 and PAX8 (Figure 3(d)). Gomori methenamine silver (GMS) stain was negative for fungal organisms. The immunohistochemical staining profile, in combination with morphology, supported the diagnosis of histiocytic sarcoma later confirmed by an expert consultation at a large center.

During the patient's subsequent hospital course, he declined rapidly and expired two weeks following admission.

## 3. Discussion

Histiocytic sarcoma (HS) is a rare neoplasm, first termed histiocytic medullary reticulosis, appearing in the medical literature in 1939 [6]. The tumors, initially considered histiocytic in origin on the basis of morphology alone, have now been shown to represent diffuse large B-cell lymphomas or peripheral T-cell lymphomas (most commonly anaplastic large cell lymphoma), by immunohistochemistry [7]. The true incidence is difficult to determine owing to its rarity but is reported to account for less than 1% of all hematolymphoid neoplasms [8]. The tumor shows bimodal age distribution with a small peak at 0–29 years and a larger peak at 50–69 years [5, 8].

The etiology and pathogenesis of HS is unknown. Common associations include midline germ cell tumors, preexisting lymphoma/leukemia, viral infection, and transplantation [5]. Due to reports of HS arising from preexisting hematopoietic malignancies, particularly in stem cell transplant patients, the concept of transdifferentiation of B-cell neoplasms to HS has developed. Molecularly complex pathways have been hypothesized to explain this phenomenon but as yet are not clearly comprehended. The disease has been documented to arise in both nodal and extranodal sites, including the gastrointestinal tract, spleen, soft tissue, and skin [7]. Solid organ presentation is not as frequent. There are only 3 published case reports in English literature that have documented the occurrence of HS postrenal transplantation (Table 1) [2–4]. One of the three cases was diagnosed within one year of transplantation and was thought to be related to Epstein-Barr virus infection [4]. In the other two reported cases and our case, HS occurred more than 10 years after renal transplantation and was not EBV-related [2, 3]. In all the three cases however, HS presented in an advanced stage as multifocal mass lesions with similar morphologic features. Involvement of bilateral native kidneys was unique to our case. No association with chronic disease of native kidneys has been reported in literature.

The incidence of malignancy following renal transplant procedures has been speculated to have a number of contributing factors including the carcinogenicity of the antirejection agents, suppression of immune surveillance mechanism, chronic antigenic stimulation, and transformation by viruses. Kramer et al. [4] described an EBV associated HS almost 3 decades back, but presently there is compelling evidence for a lack of relationship between EBV infection and an increased risk for HS [8]. Prolonged gene transcription inhibition due to long term immunosuppression with drugs like steroids and Azathioprine is hypothesized to enhance the risk of mutation in the B- and/or T-cells (translational mutations) causing differentiation into histiocytes or macrophages and subsequent proliferation of the mutated monoclonal clone [2]. Despite a very low incidence of HS in the large number of kidney transplant recipients, the role of prolonged immunosuppression as an etiology in the development of this disease is to be queried. Moreover, in our case and those reported by others [2–4], there was no documented PTLD prior to the diagnosis of HS, further challenging the theory of translational mutation. The current WHO classification does not include histiocytic sarcoma as a form of PTLD [5].

The diagnosis of HS is based on morphology supported by an extensive immunophenotypic analysis to establish histiocytic lineage and the exclusion of other, poorly differentiated, large cell malignancies [9]. The main differential diagnosis when encountering a case of HS includes Langerhans cell histiocytosis, dendritic cell sarcoma, diffuse large B-cell lymphoma, anaplastic large T-cell lymphoma, myeloid sarcoma/AML, undifferentiated carcinoma, and malignant melanoma [7–9]. The consistently similar morphologic findings described in literature can assist a pathologist in suspecting HS at the time of first encounter either on cytology or on needle core biopsy as in our case. Moreover, the morphologic features also aid in making the distinction from reactive histiocytic proliferations. This tumor is characterized by mainly dissociated single, large neoplastic cells, one or more large pleomorphic nuclei, prominent nucleoli, and abundant eosinophilic to vacuolated cytoplasm. Although hemophagocytosis is classically described in HS, more recent case series found that it is only a feature of a subset of cases [7, 9]. Our touch preps of the needle core and the needle core biopsies showed all the above features including prominent hemophagocytosis. We did not however see obvious nuclear grooves or indentations, described by others [7].

Nonspecific findings on electron microscopy and lack of universal genetic markers for detection of clonal histiocytic proliferation highlight the importance of immunohistochemistry in the diagnosis of HS [7]. A strict criterion is that the neoplastic cells must express at least two specific macrophage-associated antigens and typically lack of B-cell and T-cell markers and Langerhans cell (CD1a, langerin/CD207), follicular dendritic cell (CD21, CD23, CD35, and CAN.42), and epithelial (pancytokeratin, EMA), melanocytic (HMB-45, Melan A), and myeloid cell (CD13, CD33, myeloperoxidase) markers has been proposed to diagnose rare cases of bona fide histiocytic tumor [7–9]. Potential pitfalls include occasional expression of CD45 and CD4. Langerhans cell markers CD1a and S100 and the follicular dendritic cell marker podoplanin (D2-40) are expressed by a subset of HS [7, 8]. CD163, a hemoglobin scavenger receptor, has been recognized as a new macrophage-related differentiation marker, with higher specificity for histiocytic origin in comparison to other histiocytic markers such as CD68 [10]. More recently, T-cell immunoglobulin mucin 3 and T-cell immunoglobulin mucin 4 (TIM-3 and TIM-4) were described to be markers of histiocytic and dendritic neoplasms; however, due to their expression on dendritic cell neoplasms, Langerhans cell histiocytosis, and cases of acute monocytic leukemia, they might not be ideal to confirm HS [1].

There are no guidelines or established standard of care for treatment of HS. For historical reasons, principally misdiagnosis of non-Hodgkin lymphomas as HS, lymphoma-directed therapy such as CHOP-like regimens has been used despite a lack of data for superiority over histiocytic-directed therapies. Outcomes have thus far been poor with multifocal disease, with nearly all patients reported to experience local or distant recurrence of disease within months following therapy. Tomlin et al. [3] recently reported complete remission of postrenal transplantation HS with cladribine, high dose cytarabine, G-CSF, and Mitoxantrone followed by allogenic hematopoietic stem cell transplantation, thereby demonstrating that histiocyte-directed chemotherapy was superior to lymphoma-directed regimens. Patient died nine months after allogenic hematopoietic stem cell transplantation from bacterial pneumonia. Survivability from HS depends on stage at diagnosis, localization of the tumor, and tolerance and acceptability of the targeted therapy.

HS occurring after solid organ transplant has been reported rarely in the literature and raises the possibility of HS as a treatment-related neoplasm or a posttransplantation neoplastic disorder in solid organ transplant recipients. The diagnosis of this rare aggressive neoplasm must be suspected in the presence of an undifferentiated large cell neoplasm with hemophagocytosis and evidence of histiocytic lineage by immunohistochemistry.

## Figures and Tables

**Table 1:** Summary of cited cases of HS in renal transplant recipients.

	Age	Primary renal pathology	Duration after transplantation/treatment type	CT scan findings	Native kidney involvement	Prognosis
Aguiar et al. [2]	56/woman	HCV, CKD	28 years/azathioprine and prednisone, mycophenolate	Multiple thoracic, axillary, pelvic, and abdominal mass	Uninvolved	Died three months after diagnosis
Tomlin et al. [3]	33/man	Glomerulonephritis	Duration not specified/tacrolimus and prednisone	Multiple supraglottic and tonsillar masses with involvement of cervical lymph nodes, subcutaneous nodules in hip and thigh	Uninvolved	Died 9 months after diagnosis
Kramer et al. [4]	23/woman	Congenital anomalies, CPN	12 months/azathioprine and low dose prednisone	Multiple supra- and infratentorial brain masses, ovary, and leg	Uninvolved	Died 4 weeks after diagnosis
Our case	57/man	Hypertensive nephrosclerosis	18 years/prednisone, mycophenolate, and cyclosporine	Liver, retroperitoneal mass	Involved	Died 2 weeks after diagnosis
